# Dynamic immune and molecular responses to chronic heat stress in blood and peripheral blood mononuclear cells of dairy cows

**DOI:** 10.3389/fimmu.2025.1633453

**Published:** 2025-09-25

**Authors:** Franziska Koch, Torsten Viergutz, Christa Kühn, Björn Kuhla

**Affiliations:** ^1^ Institute for Farm Animal Biology (FBN), Dummerstorf, Germany; ^2^ Friedrich-Loeffler-Institute, Greifswald, Germany

**Keywords:** hyperthermia, hematology, NF-κB p65, coagulation, platelets

## Abstract

**Background:**

As a result of climate change, dairy cows even in confinement systems are exposed to high ambient temperatures and environmental factors inducing heat stress. However, there are indications that chronic heat stress with reduced feed intake initially stimulates a systemic inflammatory response and progressively reduces the immunocompetence. This finally increases the disease susceptibility. This study aimed to elucidate the effects of chronic heat stress or equivalent reduced feed intake via pair-feeding at thermoneutrality on the daily hematological profile, leukocyte NF-κB p65 signaling pathway, immune function, and metabolism in peripheral blood mononuclear cells (PBMC) of dairy cows.

**Methods:**

Primiparous, mid-lactating Holstein cows (*n* = 30) were assigned to heat-stressed (HS; temperature-humidity index (THI) 76, 28°C, relative humidity (RH) 50%), control (CON; THI 60, 16°C, RH 69%), or pair-fed (PF; THI 60, 16°C, RH 69%) group for 7 days.

**Results:**

HS cows showed a lower number of erythrocytes, platelets, lymphocytes, hemoglobin, hematocrit, and iron concentration and increased endotoxin concentration compared to PF cows. The presence of NF-κB p65 in the nucleus of leukocytes was lower in HS than in the two other groups on day 5, while it was higher in HS cows on day 6. Furthermore, on day 6, RNA sequencing of PBMC showed an enrichment of higher expressed genes in pathways of platelet activation, coagulation cascade, leukocyte transendothelial migration, and focal adhesion in HS cows compared to both non-heat-stressed groups. However, T cell receptor signaling pathway, intestinal immune network for IgA production, antigen processing and presentation, and metabolic processes were expressed lower in HS compared to CON cows.

**Conclusion:**

The results suggest that heat stress induces dynamic changes of the red blood cell and lymphocyte profiles but only transiently activating the leukocytic NF-κB p65 signaling pathway while suppressing T cell signaling, both likely in response to the increased circulating endotoxin concentration. The activation of platelets and the coagulation cascade were likely due to heat-stress-induced microvascular injuries, altered hematocrit, or vasodilatation. Altered blood coagulation and immune responses need to be considered in the management of heat-stressed dairy cows.

## 1 Introduction

Climate change is expected to enhance the frequency of extreme weather events such as heat waves in the coming years (1). High ambient temperatures and humidity during summer months are a thermal threat not only for humanity (2) but also for farm animals, jeopardizing their well-being and welfare (3). Under these climatic conditions, animals adapt to environmental heat by decreasing internal heat production and increasing heat loss (4). Particularly, dairy cows are prone to heat stress and lose their ability to disperse endogenously produced heat when exceeding the thermoneutral zone (5).

The blood circulation facilitates the heat transfer from the internal organs to the outer surface by directing the blood flow toward the skin and promoting vasodilatation (6–8). Another strategy is minimizing endogenous heat production by reducing feed intake, which, however, also alters the milk quantity and quality (9, 10). Furthermore, there is rising evidence that heat stress has multiple negative effects on the physiological performance of blood cells as indicated by a lower red blood cell (RBC) count (11) and hematocrit level (11, 12), potentially reducing the total oxygen binding capacity (13). Dairy cows exposed to high ambient temperatures in the summer also had a lower number of lymphocytes than in winter time, indicating an altered immune function during heat stress (14). The causes for the reduced lymphocyte number could be attributed to a reduction of lymphocyte proliferation and DNA synthesis rate (15). An impaired immune system facilitates the susceptibility of animals to inflammatory processes (16–19), the incidence of diseases (e.g., laminitis, mastitis, metritis) (20–23), and metabolic dysfunctions (e.g., displaced abomasum, acidosis) (24, 25) during heat stress.

On the molecular level, the heat-stress-mediated impaired immune function is characterized by the activation of various signal transduction pathways altering the gene expression of immune cell mediators and cytokines without signs of an infection (26–28). It is assumed that a summary of metabolic stress and heat stress stimulate the inflammatory response and that these challenging events may progressively reduce the immunocompetence and increase the susceptibility to inflammation (27). Among the stressed tissues is the intestinal tract, which, as a “leaky gut” (29), allows toxic particles and bacteria to infiltrate the organism during heat stress (30). The bacterial particles are captured by heat shock proteins or by lipopolysaccharide-binding protein (LBP) and present to the soluble or membrane-bound CD14 receptor on monocytes or macrophages (31). This activates the translocation of nuclear factor kappa-light-chain-enhancer of activated B cells p65 (NF-κB p65) from the cytosol to the nucleus. Macrophages and monocytes in the gut receive signals via toll-like receptors (TLR) 2 and 4, thereby promoting the production of pro-inflammatory cytokines, e.g., tumor necrosis factor α (TNFα), interleukin-1β, and interferon γ (INFγ) (32). In an earlier study, we could detect very early signs of inflammation in the systemic circulation and the local mesenteric lymph nodes of heat-stressed (HS) dairy cows that were exposed to 28 °C for 7 days (33). These HS cows showed higher plasma haptoglobin, TNFα, and INFγ concentrations and a higher abundance of *TNFA* and *INFG* mRNA as well as a tendency for higher TLR2 protein expression in mesenteric lymph nodes (33). However, limited information is available on a potential direct induction of the CD14/TLR2/4/NF-κB p65 signaling in blood leukocytes to heat stress and reduced feed intake in lactating cows.

In this study, we hypothesize that heat stress stimulates the gene expression of pro-inflammatory cytokines in blood monocytes via the NF-κB p65 translocation to the nucleus contributing to disturbed immunity. In order to consider potential indirect metabolic stress effects, we utilized pair-feeding as an additional control group to mimic a reduced nutrient availability but under thermoneutral conditions to distinguish between heat and metabolic insults. Furthermore, we utilized a holistic approach to study the immune and metabolic function in isolated peripheral blood mononuclear cells (PBMC) by RNA sequencing to provide new insights into the molecular mechanism of cows when coping with heat stress. Therefore, our objective was to elucidate the effects of chronic HS and PF at thermoneutrality on the daily white and red blood cell count, pro-inflammatory cytokine gene expression in leukocytes and CD14^+^ cells, nuclear protein expression of NF-κB p65 in leukocytes, and RNAseq of PBMC at two time points (1 day and 6 days of treatment) in primiparous lactating dairy cows.

## 2 Materials and methods

### 2.1 Animal selection and treatment

The present study was part of a larger project described earlier (33). Briefly, 30 primiparous, non-pregnant German Holstein cows (169 ± 48 days in milk (DIM)) were evenly allocated to heat-stressed (HS, *n* = 10), control (CON, *n* = 10), or pair-fed group (PF, *n* = 10). All cows were adapted to the climate chamber at thermoneutral conditions (permanent 16°C and temperature–humidity index (THI) = 60) for 6 days and received a total mixed ratio *ad libitum* twice daily at 0730 h and 1730 h (adaptation phase) (33). In the experimental phase, HS cows were kept for seven days at 28°C with 51% ± 2% relative humidity (RH), resulting in a THI of 76 with *ad libitum* feeding and water access. The CON group was exposed for seven days to 16°C with 69% ± 2% RH and a THI of 60 with *ad libitum* feeding. The PF cows, as the second control group to mimic metabolic stress, were exposed for seven days to 16°C with 69% ± 2% RH and a THI of 60. In order to ensure isoenergetic and isonutritive feeding of HS and PF cows, the feed intake of HS cows was calculated as a percentage of the daily mean feed intake per kilogram of body weight (33). In the climate chambers, the day–night rhythm was given by a light cycle ranging from 0600 to 1900 h. The cows were milked at 0700 h and 1730 h daily. The animal studies were approved by the ethics committee of the State Government in Mecklenburg-West Pomerania, Germany (LALLF M-V/TSD/7221.3-1.1-60/19). The studies were conducted in accordance with the local legislation and institutional requirements. Written informed consent was obtained from the owners for the participation of their animals in this study. All methods were in compliance with the ARRIVE guidelines (34).

### 2.2 Blood sampling and PBMC isolation

At 1 day before the start of the experimental phase, the cows were equipped with an indwelling jugular catheter (Cavafix, B. Braun, Melsungen, Germany). Blood samples were taken after feeding in the morning according to the following scheme: 0 h, 6 h, day 1, day 2, day 3, day 4, day 5, day 6, and day 7, each into 9-mL monovettes (Sarstedt, Nümbrecht, Germany) containing EDTA or into 9-mL serum tubes and immediately cooled on ice. After collection, the EDTA blood samples were centrifuged at 0.7 × *g* for 20 min at 4°C. The serum samples were kept at 4°C overnight and centrifuged using the same conditions. The plasma and serum samples were aliquoted and stored at -80°C until further analysis. On day 1 and day 6, additional 4-mL blood samples were subjected to PBMC isolation utilizing a Histopaque 1077 gradient (Sigma-Aldrich, Schnelldorf, Germany) (35). The isolated PBMC were frozen in liquid nitrogen and stored at -80°C before further analysis.

### 2.3 Complete blood count

Whole-blood samples from EDTA-coated tubes were instantaneously subjected to a complete blood count using a hematology analyzer VetScan HM5 (Scil Animal Care Company, Vierheim, Germany). The total white blood cell count (WBC), WBC differential counts for neutrophils, eosinophils, basophils, lymphocytes, and monocytes, as well as the red blood count (RBC), hematocrit value (HCT), hemoglobin concentration (HGB), mean corpuscular volume (MCV), mean corpuscular hemoglobin (MCH), mean corpuscular hemoglobin concentration (MCHC), and RBC distribution width (RDW) were analyzed. In addition, platelet indices were analyzed, including the platelet count, mean platelet volume (MPV), platelet distribution width (PDW), and plateletcrit (PCT).

### 2.4 Serum metabolites

In serum samples, total iron concentrations were measured spectrophotometrically and potentiometrically (ABX Pentra C400 clinical chemistry analyzer; HORIBA Europe GmbH, Oberursel, Germany) using a commercial kit (A11A01-637; HORIBA ABX SAS, Montpellier, France). Serum LPS-binding protein (LBP) concentrations were measured by a commercially available ELISA (HK503, Hyltec Biotech). The intra- and inter-assay coefficients of variation were 7.2% and 15.5%, respectively.

### 2.5 Endotoxin

Circulating Gram-negative bacterial endotoxin concentrations were determined by a limulus amebocyte lysate (LAL) chromogenic endpoint assay using serum samples (HIT302, Hycult Biotech, Uden, Netherlands) according to manufacturer’s instructions. The assay was performed using certified endotoxin-free and depryogenated consumables in a sterile bench. The standards and samples were diluted in glass tubes incubated for 4 h at 200°C. The control standard endotoxin was diluted 1:10 with endotoxin-free water for serial dilutions, with final standard concentrations of 10, 4, 1.6, 0.64, 0.26, 0.10, and 0.04 EU/mL and blank. The samples were diluted 1:10 and assessed in duplicates on a 96-well plate. A reconstituted LAL reagent was prepared according to the individual kit certificate. The samples and standard (50 µL) were mixed with 50 µL LAL reagent, sealed, and incubated at room temperature for 20 min. Subsequently, the absorption was measured every 5 min at 405 nm until the difference between 10 and 4 EU/mL was ≤10%. Finally, 50 µL of stop solution was added, and the sample mixture was measured at 405 nm in a plate reader (Tecan infinite 200, Männedorf, Switzerland). The final sample’s endotoxin concentration was calculated against the absorbance of the standards in a linear regression model including the elimination of the background signal from the blank. Serum samples of the same experimental day were measured on the same plate. The intra- and inter-assay coefficients of variation were 6.0% and 17.3%., respectively.

### 2.6 Immunofluorescence staining for nuclear NF-κB p65

The protocol for measuring the presence of NF-κB in the nucleus was adapted from Selkirk et al. (36). For each sampling time point (except for 6 h), duplicates of 50 µL of whole blood were incubated with 500 µL VersaLyse (Beckman Coulter, Krefeld, Germany) for 20 min. The samples were centrifuged for 5 min at 250 × *g*, and the supernatant was discarded. The cell pellets were washed with 500 µL PBS (Gibco Life Technologies, Carlsbad, CA, USA) and centrifuged for 5 min at 150 × *g*, and the supernatant was discarded. For nuclei isolation, BD CycleTest Plus DNA reagent kit was used according to the manufacturer’s instructions (BD Bioscience, Franklin Lakes, NJ, USA). In brief, the cell pellet was re-suspended in 500 µL of citrate buffer and centrifuged for 5 min at 300 × *g*, the supernatant was discarded, and the washing with citrate buffer was repeated. Thereafter, 125 µL of solution A was added and incubated 10 min in the dark, following the incubation with 100 µL of solution B for 10 min in the dark. Isolated nuclei were stained with 1.1 µL NF-κB p65 antibody (C22B4, rabbit monoclonal antibody, Cell Signaling Technology, Denvers, MA, USA), and the duplicate treated without primary antibody served as the negative control. After 30 min of incubation, 2.2 µL goat anti-rabbit AlexaFlour488 (1:10; Invitrogen, Thermo Fisher Scientific) was added and incubated for 30 min at 4°C. Solution C (100 µL) was added and incubated for 10 min at 4°C. The samples were quantified on a Gallios flow cytometer (Beckman Coulter) and analyzed using Kaluza software (Beckman Coulter). For the intra-nuclear NF-κB analysis, double-gating on a forward vs. side scatter dot plot and FL2 PI staining were used to analyze 10,000 cells. Flow cytometry immunofluorescence acquisition was performed within 1 h of staining. To calculate the NF-κB p65 fluorescence intensity, the fluorescence intensity of the negative control was subtracted from each NF-κB p65-stained sample.

### 2.7 Immunofluorescence staining for HSP70

For each sampling time point, 100 µL of whole blood was incubated for 20 min with 1,000 µL VersaLyse (Beckman Coulter). The samples were centrifuged for 5 min at 250 × *g*, and the supernatant was discarded. The cell pellets were washed twice with 700 µL 1× PBS (Gibco Life Technologies). Subsequently, the cell pellet was dissolved in 100 µL PBS and frozen in ice-cold methanol (VWR International, Darmstadt, Germany) for further analysis. Methanol-fixed cells were washed twice with 1× PBS. For staining, duplicates of each time point were prepared to either stain with HSP70 (SMQ-SMC-100B, mouse monoclonal antibody, Biozol, Sontheim an der Brenz, Germany) or without antibody as negative control and incubated overnight at 4°C. On the next day, the cells were washed with 1× PBS, and duplicates were stained with rabbit anti-mouse AlexaFlour488 antibody (1:1,000, Thermo Fisher Scientific, Waltham, MA, USA) with incubation for 1 h at 4°C. Thereafter, the cells were washed with 1× PBS, resuspended in 500 µL 1× PBS containing Hoechst 33342 (0.5 µM final concentration, Invitrogen, ThermoFisher), and incubated for 20 min at 4°C. The samples were quantified on a Gallios flow cytometer (Beckman Coulter) and analyzed using Kaluza software (Beckman Coulter). The mean fluorescence intensity of the negative control was subtracted from each HSP70-stained.

### 2.8 PrimeFlow^®^ RNA assay

The PrimeFlow^®^ RNA assay (Invitrogen, Thermo Fisher Scientific) was performed daily on blood samples following the manufacturer’s protocol divided over 2 days. All buffers were included in the PrimeFlow™ RNA assay kit, and specific target probe sets for bTNFA:A488 and bIL1B:A647 (target set 1) and bIL6:A488 and bIFNG:A647 (target set 2) were designed and purchased from Thermo Fisher Scientific. Cell surface staining was performed in duplicate each by incubating 100 µL blood with 10 µL CD14:AlexaFluor700 (MCA1568A700, BioRad, Hercules, CA, USA) for 30 min at 4°C. Subsequently, the cells were fixed for 30 min at 4°C. After permeabilization, the cells were incubated for a second time with fixation buffer for 1 h at room temperature in the dark. Then, the cells were incubated for the hybridization with the appropriate target probe sets for 2 h at 40°C. The samples were kept overnight at 4°C in the dark. On the following day, pre-amplification and amplification of the hybridization were performed twice for 1.5 h at 40°C with the pre-amplification mix and subsequently the amplification mix. Next, the cells were incubated with the label probe set for 1 h at 40°C and quantified thereafter on a flow cytometer (Gallios, Beckman Coulter). The analysis was performed with Kaluza software (Beckman Coulter) based on total cell count and percentage of CD14^+^ cell surface staining.

### 2.9 Transcriptome analysis by RNA sequencing

Isolated PBMC obtained on days 1 and 6 were utilized to extract RNA by utilizing the NucleoSpin RNA mini kit (Macherey-Nagel, Düren, Germany) with an additional DNase digestion step (37). The extracted RNA was tested for genomic DNA contamination by performing PCR (38). RNA concentration was measured with a NanoDrop 2000 spectrophotometer (Thermo Fisher Scientific), and quality was verified by using a Qubit 2.0 fluorometer (Thermo Fisher Scientific). RNA integrity was evaluated by utilizing the Bioanalyzer 2100 (Agilent Technologies, Böblingen, Germany) with mean RIN values of 8.36 (min 7.6 and max 9.3). Subsequently, a stranded library preparation protocol for RNA sequencing was applied (TruSeq mRNA sample prep kit, Illumina, San Diego, CA, USA) using indices for multiplexing and polyA selection to focus on polyadenylated RNA (in the majority mRNA). The RNAseq libraries were checked for quality on the Bioanalyzer 2100 and subjected to paired-end sequencing (2 × 100 bp) using the Illumina HiSeq 2500 system (Illumina).

### 2.10 Bioinformatic analysis

The CASAVA (Illumina) software was used for demultiplexing of reads. Scripts written in Linux [including command from the samtools suite (39)] and R were applied for data processing. For quality control, read alignment, and transcript quantification, we used the nf-core/rnaseq -r 3.4 pipeline. This included removing adapters and low-quality bases with Cutadapt version 3.4. The reads were aligned to the bovine reference genome ARC-UCD 1.2 with Ensembl reference annotation version 105 using STAR version 2.6.1d. In the nf-core/rnaseq pipeline, the option to use Salmon version 1.5.2 was selected to establish expression counts at the gene level. Differential expression analysis was performed with DESeq2 version 1.26.0 (40), with a threshold for significance of adjusted *P* (Padj) <0.05.

### 2.11 Functional enrichment analysis

The Database for Annotation, Visualization, and Integrated Discovery (DAVID, version 6.8 with updates from December 2021) was utilized to investigate enriched biological themes and cluster analysis (41). The unique list of differentially expressed genes with ensemble gene ID (*q* < 0.05) was submitted as gene list and *Bos taurus* database as background. The cutoff value for multiple enrichment testing determined via the Benjamin–Hochberg algorithm was 0.05. Only the results from Gene Ontology (GO) analysis and Kyoto Encyclopedia of Genes and Genomes (KEGG) pathway analysis were selected for functional annotation categories (https://david.ncifcrf.gov/tools.jsp).

### 2.12 Statistical analysis

Daily measurements on the same animal were analyzed by repeated-measurement ANOVA using the MIXED procedure of SAS (Version 9.4, SAS Institute Inc., Cary, NC, USA). Based on Akaike’s Information Criteria (AIC), the autoregressive type or compound symmetry for the block diagonal residual covariance matrix was chosen. The model contained the fixed effects of treatment (HS, CON, PF), time (experimental day), the interaction (treatment × time), and days in milk which served as covariate. Least squares means (LSM) and their standard errors (SE) were computed for each fixed effect in the ANOVA model. Additionally, differences of these LSM were tested using the Tukey–Kramer procedure. The SLICE statement of PROC MIXED was used to perform a partitioned analysis of the LSM for the interaction treatment × time. Results were considered as statistically significant at *P <*0.05 and tendencies at 0.05 < *P <*0.09.

## 3 Results

### 3.1 Complete blood count

As shown in **Table 1**, the number of erythrocytes was significantly lower in HS than PF cows on day 7 (*P* < 0.05). The hemoglobin concentration (*P* = 0.07) and hematocrit (*P* = 0.09) parameters tended to be lower in HS than PF cows on day 7. Furthermore, there was a significant treatment × day interaction for the number of erythrocytes (*P* < 0.01), hemoglobin concentration (*P* < 0.01), and hematocrit (*P* < 0.001). In addition, the RDW was significantly lower in HS than PF cows on day 0, 6 h, and day 2 (*P* < 0.05, respectively) and significantly lower in HS and CON than PF cows between days 4 to 7 (*P* < 0.05, respectively). No group differences were observed for the MCV, MCH, and MCHC values.

**Table 1:** Red blood cell count and serum iron concentration in heat-stressed (HS), control (CON), or pair-fed (PF) dairy cows (*n* = 10 cows per group).

		Days										*P*-value		
Parameter	Treatment	0 h	6 h	1	2	3	4	5	6	7	SEM^a^	Treatment	Day	Treatment × day
Erythrocytes, ×10^12^ cells	HS	6.97	6.93	6.93	6.91	6.91	6.85	6.74	6.79	6.47b	0.18	0.497	<0.01	<0.01
	CON	6.98	6.99	7.08	7.00	7.02	7.09	6.85	7.00	6.81a,b	0.18			
	PF	7.03	6.82	6.92	7.12	7.20	7.39	7.23	7.19	7.09a	0.18			
HGB, g/dL	HS	9.48	9.37	9.40	9.20	9.19	9.14	9.06	9.12	8.74B	0.27	0.618	0.079	<0.01
	CON	9.49	9.52	9.56	9.32	9.40	9.53	9.29	9.34	9.19A,B	0.27			
	PF	9.43	9.28	9.24	9.54	9.58	9.87	9.63	9.63	9.59A	0.27			
HCT, %	HS	30.55	30.44	30.69	30.44	30.19	30.33	29.93	29.83	28.81B	0.86	0.563	<0.01	<0.001
	CON	30.57	30.57	30.78	30.50	30.76	30.93	29.94	30.70	29.79A,B	0.86			
	PF	30.57	29.73	30.00	31.19	31.72	32.96	32.31	31.87	31.39A	0.87			
Fe, µmol/l	HS	25.0	24.7	23.9	22.5B	22.0B	20.3	18.0B	18.5B	13.8b	2.5	0.094	<0.001	0.263
	CON	25.0	27.7	29.0	25.0A,B	29.0A	23.3	25.7A	24.3A,B	24.1a	2.5			
	PF	28.0	26.9	27.3	29.3A	25.7A,B	24.6	24.4A,B	26.3A	23.7a	2.5			
RDW, %	HS	22.38b	22.58b	22.58	22.44b	22.79	22.82b	22.64b	22.64b	22.86b	0.48	<0.05	<0.001	<0.01
	CON	22.95a,b	22.96a,b	22.90	22.87a,b	22.95	22.84b	23.19b	22.86b	22.98b	0.48			
	PF	24.12a	24.23a	23.90	24.45a	24.61	24.87a	24.94a	25.09a	24.97a	0.48			
MCV, fl	HS	44.25	44.25	44.66	44.46	43.97	44.38	44.59	44.40	44.81	1.23	0.935	<0.05	<0.05
	CON	43.91	43.71	43.52	43.63	44.04	43.85	43.86	43.87	44.08	1.24			
	PF	43.55	43.65	42.36	43.97	44.18	44.59	44.60	44.41	44.22	1.24			
MCH, pg	HS	13.68	13.58	13.67	13.40	13.35	13.40	13.53	13.46	13.51	0.35	0.976	<0.05	0.915
	CON	13.60	13.67	13.52	13.34	13.44	13.47	13.62	13.35	13.52	0.35			
	PF	13.44	13.68	13.33	13.36	13.29	13.40	13.33	13.38	13.55	0.35			
MCHC, g/dL	HS	31.01	30.76	30.67	30.26	30.44	30.17	30.28	30.40	30.33	0.35	0.512	<0.001	0.680
	CON	31.01	31.19	31.07	30.54	30.56	30.83	31.03	30.36	30.84	0.35			
	PF	30.92	31.35	30.79	30.54	30.18	30.01	29.85	30.24	30.59	0.35			
Platelet count, x10^9^ cells	HS	238.0	231.2	203.9	215.2	215.8	213.2	218.6	206.3	209.3	16.8	0.643	0.900	<0.05
	CON	199.3	203.1	207.8	197.4	195.5	201.7	207.8	207.2	201.2	16.8			
	PF	209.5	203.0	201.4	218.3	216.7	211.0	216.2	242.0	242.8	16.9			
Platelet count, %	HS	0.15	0.14	0.15	0.13	0.13	0.13	0.13	0.12B	0.13	0.01	0.559	0.889	<0.01
	CON	0.12	0.13	0.13	0.12	0.12	0.12	0.13	0.13A,B	0.12	0.01			
	PF	0.13	0.13	0.12	0.14	0.13	0.13	0.13	0.16A	0.15	0.01			
PCT, %	HS	0.15	0.14	0.15	0.13	0.13	0.13	0.13	0.12B	0.13	0.01	0.559	0.889	<0.01
	CON	0.12	0.13	0.13	0.12	0.12	0.12	0.13	0.13A,B	0.12	0.01			
	PF	0.13	0.13	0.12	0.14	0.14	0.13	0.13	0.16A	0.15	0.01			
MPV, fl	HS	6.17	6.05	6.29	6.15	6.12	6.10	6.13	5.97b	5.97B	0.11	0.196	0.844	<0.05
	CON	6.07	6.11	6.06	6.10	6.02	6.04	6.05	6.07b	5.99A,B	0.11			
	PF	6.25	6.29	6.12	6.30	6.28	6.37	6.14	6.47a	6.31A	0.11			
PDW, %	HS	30.59a,b	30.21a,b	31.33	30.49	30.43	29.72b	29.95	29.76b	30.13a,b	0.57	<0.05	0.913	<0.05
	CON	29.57b	30.00b	29.76	30.31	29.86	30.5a,b	29.89	30.00b	29.80b	0.58			
	PF	31.8a	32.03a	31.14	31.53	31.56	32.05a	31.24	32.22a	32.01a	0.58			

Within day, different lowercase letters indicate significant difference between groups (*P* < 0.05), and different capital letters indicate 0.05 < *P* < 0.09.

Fe, iron; fl, femtoliter; HCT, hematocrit; HGB, hemoglobin; MCH, mean corpuscular hemoglobin; MCHC, mean corpuscular hemoglobin concentration; MCV, mean corpuscular volume; MPV, mean platelet volume; PDW, platelet distribution width; PCT, plateletcrit; RBC, red blood cell count; RDW, red blood cell distribution width.

a Pooled SEM for each treatment.

All data are given as LSM ± SEM.

For the platelet parameters, neither the platelet count, percentage of platelet counts, MPV, or plateletcrit reached the overall significance level for treatment (**Table 1**). However, the percentage of platelet counts and plateletcrit tended to be lower in HS than PF cows on day 6 (*P* < 0.08), yet the MPV was (*P* < 0.05) or tended to be (*P* = 0.08) lower in HS than PF cows on days 6 and 7, respectively. In addition, the PDW was significantly lower in HS than PF cows on days 4 and 6 (*P* < 0.05, respectively) and was significantly higher in PF than CON cows on day 0, 6 h, and days 6 and 7 (*P* < 0.05).

The number of blood lymphocytes in HS cows was (*P* < 0.05) or tended to be (*P* = 0.08) lower than in PF cows between days 3 and 7 (**Table 2**). During the same time, the number of lymphocytes was or tended to be higher in PF compared to CON cows (*P* < 0.05, respectively). In addition, there was a significant treatment × day interaction for lymphocytes (*P* < 0.05) and a tendency for the eosinophils (*P* = 0.08) and the percentage of neutrophils (*P* = 0.094). However, no differences were found among the groups for the percentage and the number of leukocytes, monocytes, neutrophils, eosinophils, and basophils.

**Table 2:** White blood cell count in heat-stressed (HS), control (CON), and pair-fed (PF) dairy cows (*n* = 10 cows per group).

		Days										*P*-value		
Parameter	Treatment	0 h	6 h	1	2	3	4	5	6	7	SEM^a^	Treatment	Day	Treatment × day
Lymphocytes, ×10^9^ cells/l	HS	5.05	5.22	5.02	5.16	5.08b	5.41a,b	5.36a,b	5.28A,B	4.63B	0.30	0.101	<0.05	<0.05
	CON	5.06	5.25	5.20	5.20	5.04b	4.91b	4.76b	4.80B	4.69B	0.30			
	PF	5.63	5.56	5.65	5.70	6.14a	6.02a	5.94a	5.75A	5.60A	0.30			
Leukocytes, ×10^9^ cells/l	HS	8.91	9.47	8.88	9.38	9.59	10.01	9.85	9.61	8.33	0.58	0.300	<0.001	0.816
	CON	8.95	9.76	9.19	9.49	9.23	10.25	9.53	8.79	8.46	0.58			
	PF	10.53	10.59	10.42	9.88	10.31	10.55	10.46	9.92	9.50	0.58			
Monocytes, ×10^9^ cells/l	HS	0.17	0.24	0.21	0.32	0.31	0.31	0.22	0.22	0.27	0.10	0.597	0.087	0.475
	CON	0.29	0.36	0.25	0.25	0.19	0.43	0.38	0.24	0.17	0.10			
	PF	0.37	0.27	0.28	0.28	0.44	0.55	0.35	0.39	0.29	0.10			
Neutrophils, ×10^9^ cells/l	HS	3.42	3.79	3.39	3.65	3.89	3.94	3.94	3.73	3.11	0.44	0.953	<0.05	0.438
	CON	3.38	3.90	3.52	3.80	3.77	4.64	4.16	3.47	3.37	0.44			
	PF	4.12	4.39	4.17	3.60	3.43	3.67	3.81	3.41	3.35	0.44			
Eosinophils, ×10^9^ cells/l	HS	0.21	0.18	0.21	0.20	0.25	0.28	0.27	0.31	0.26	0.04	0.486	<0.05	0.080
	CON	0.18	0.19	0.17	0.20	0.19	0.22	0.20	0.23	0.19	0.04			
	PF	0.28	0.30	0.27	0.24	0.24	0.25	0.28	0.29	0.21	0.04			
Basophils, ×10^9^ cells/l	HS	0.05	0.05	0.05	0.05	0.06	0.07	0.07	0.07	0.06	0.01	0.408	0.063	0.380
	CON	0.04	0.04	0.04	0.04	0.04	0.05	0.05	0.05	0.04	0.01			
	PF	0.07	0.07	0.06	0.06	0.05	0.06	0.07	0.08	0.06	0.01			
Lymphocytes, %	HS	57.08	55.50	57.03	55.14	53.36	54.69	49.64	56.12	55.88	2.82	0.711	0.150	0.230
	CON	57.26	55.91	56.94	55.64	54.64	49.31	53.31	55.19	56.80	2.83			
	PF	54.79	52.92	54.88	58.02	59.88	57.02	57.25	58.28	59.25	2.84			
Monocytes, %	HS	1.90	2.54	2.25	3.24	3.05	2.91	2.21	2.10	3.24	0.92	0.663	0.170	0.413
	CON	2.88	3.26	2.50	2.54	1.99	4.22	4.02	2.57	1.98	0.92			
	PF	3.40	2.61	2.72	2.90	4.13	5.24	3.33	3.83	2.93	0.93			
Neutrophils, %	HS	38.03	39.51	37.59	38.84	40.42	38.88	38.91	37.59	36.93	2.60	0.527	0.560	0.094
	CON	37.24	38.21	38.11	39.13	40.55	43.76	41.99	39.01	38.42	2.60			
	PF	38.40	41.00	39.19	36.02	33.13	34.81	36.03	34.11	35.04	2.61			
Eosinophils, %	HS	2.40	1.97	2.51	2.19	2.54	2.84	2.71	3.43	3.18	0.51	0.762	<0.01	0.132
	CON	2.15	2.13	1.98	2.20	2.14	2.17	2.13	2.63	2.30	0.51			
	PF	2.74	2.81	2.61	2.50	2.31	2.43	2.76	3.02	2.22	0.51			
Basophils, %S	HS	0.61	0.52	0.64	0.58	0.66	0.72	0.72	0.80	0.77	0.13	0.574	<0.01	0.334
	CON	0.48	0.47	0.48	0.51	0.47	0.51	0.51	0.60	0.49	0.13			
	PF	0.68	0.66	0.60	0.61	0.53	0.52	0.66	0.80	0.61	0.13			

Within day, different lowercase letters indicate significant difference (*P* < 0.05) between groups, and different capital letters indicate 0.05 < *P* < 0.09.

a Pooled SEM for each treatment.

All data are given as LSM ± SEM.

### 3.2 Serum iron, endotoxin, and LBP

**Figure 1:**
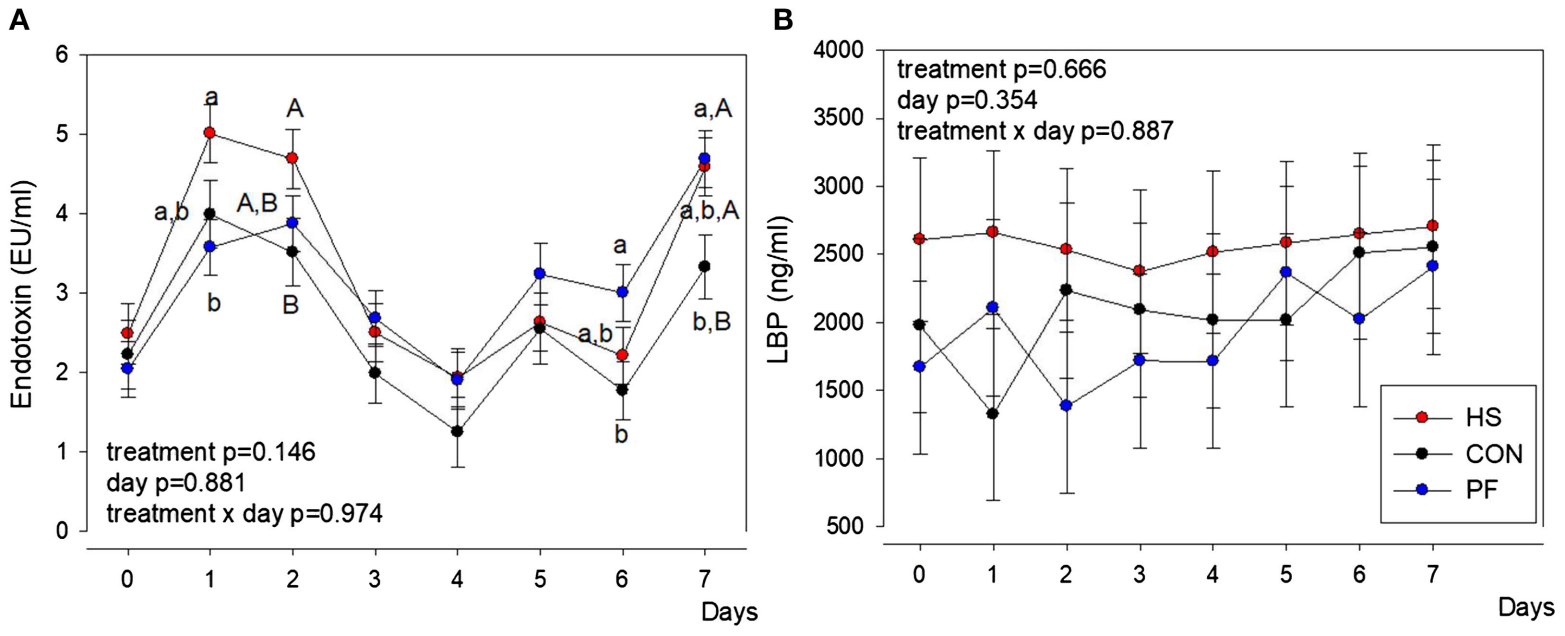
Changes in **(A)** serum endotoxin and **(B)** serum LPS-binding protein (LBP) concentrations in heat-stressed (HS), control (CON), and pair-fed (PF) dairy cows. During the 7-day experimental phase, HS cows (red) were kept at an ambient temperature of 28 °C (THI 76), and PF (blue) and CON (black) cows were exposed to 16°C and THI 60 for 7 days. Serum endotoxin concentrations fluctuate dynamically and are or tended to be greater in HS than PF cows on days 1 and 2. *n* = 10 cows per group. All data are given as LSM ± SEM. Different lowercase letters indicate *P* < 0.05, and different uppercase letters indicate 0.05 < *P* < 0.09.

The HS cows tended to have lower serum iron concentrations than PF or CON cows (*P* < 0.1), but on day 7, the serum iron concentration was significantly lower in HS than CON and PF cows (*P* < 0.05; **Table 1**). The serum endotoxin concentration was not significantly different between groups but higher in HS than PF cows on day 1 and tending to be higher in HS than CON cows on days 2 and 7 (*P* < 0.09, respectively; **Figure 1A**). Furthermore, PF cows showed higher endotoxin concentrations in comparison to CON cows on days 6 and 7. However, the concentration of the acute-phase protein LBP was not affected by treatment (**Figure 1B**).

### 3.3 Nucleic NF-κB p65 abundance

**Figure 2:**
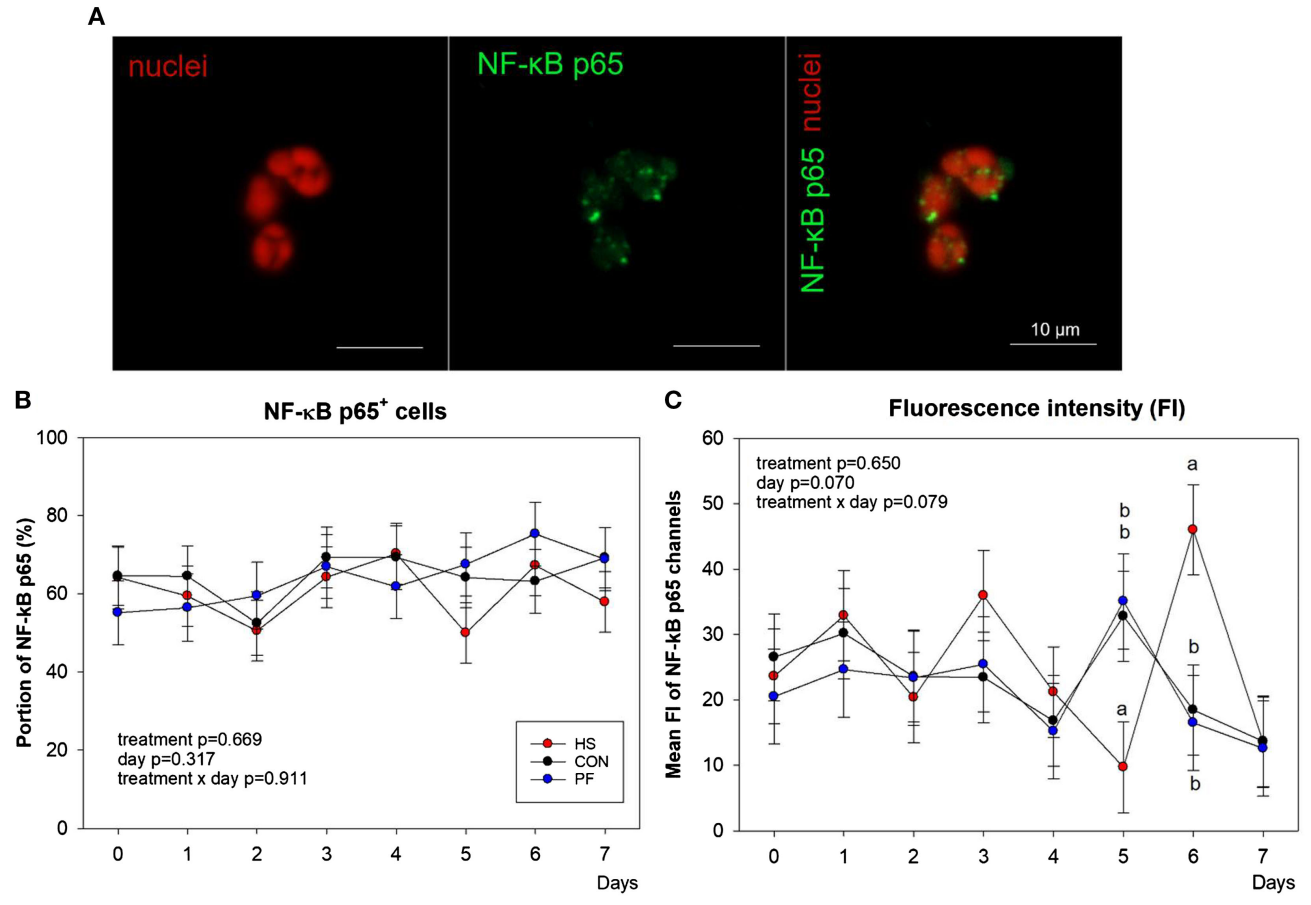
Flow cytometry analysis of nuclear factor kappa-light-chain-enhancer of activated B cells p65 (NF-κB p65) positive blood leukocytes intensity displayed a dynamic response, decreasing on day 5 in heat-stressed (HS) cows and subsequently rising on day 6 in comparison to the control (CON) and pair-fed (PF) cows. **(A)** Representative fluorescence microscopy picture of nuclei (red) and NF-κB p65 (green) staining and overlay. Bars represent 10 µm. **(B)** Portion of NF-κB p65 positive leukocytes and **(C)** mean fluorescence intensity (FI) of NF-κB p65 in heat-stressed (HS, red), control (CON, black) feeding, and pair-fed (PF, blue) dairy cows. *n* = 10 cows per group. All data are given as LSM ± SEM. Different lowercase letters indicate *P* < 0.05.

To evaluate if the temporary higher endotoxin concentrations in HS cows triggered NF-κB p65 translocation into the nucleus of leukocytes, fluorescence double-labeling and subsequent flow cytometry analyses were performed (**Figure 2A**). The portion of NF-κB p65 positively stained nuclei did not differ by treatment or time (**Figure 2B**). However, the mean fluorescence intensity (FI) of NF-κB p65 labeling was significantly lower in HS than CON and PF cows on day 5 (*P* < 0.05), whereas on day 6, it was significantly higher in HS than CON and PF cows (*P* < 0.05, **Figure 2C**). Interestingly, heat stress did not affect the leukocytic HSP70 protein expression as determined by fluorescence intensity (**Supplementary Figure S2**).

### 3.4 Cytokine mRNA abundances

**Figure 3:**
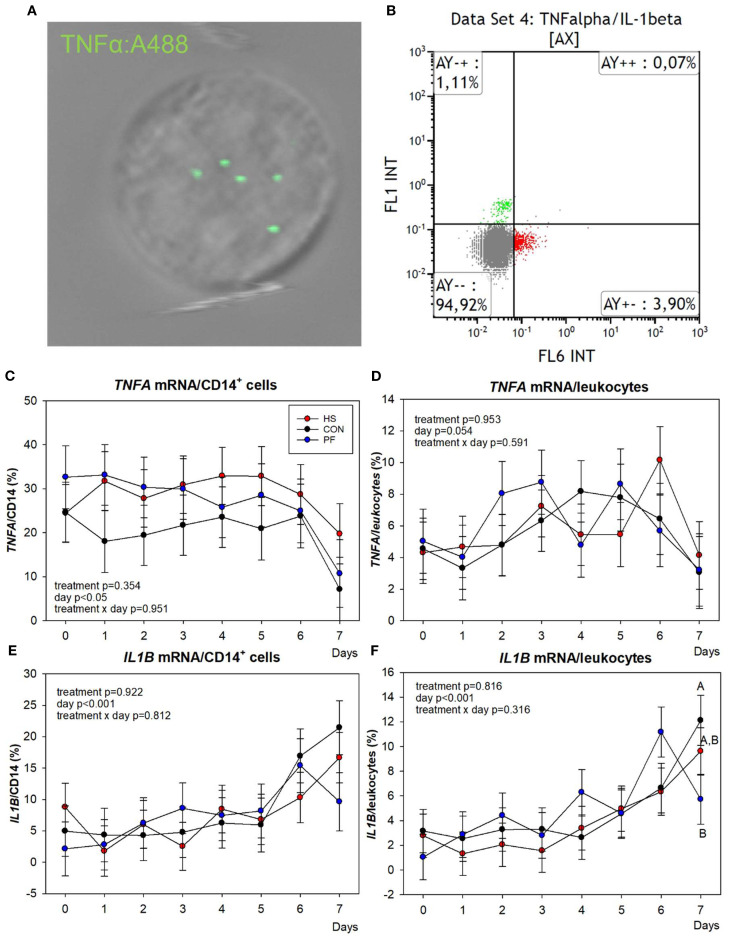
*TNFA* and *IL1B* mRNA abundance in blood leukocytes of heat-stressed (HS, red), control (CON, black), and pair-fed (PF, blue) cows by PrimeFlow RNA assay. The leukocytes were stained with antibodies for CD14, fixed, permeabilized, and hybridized with gene probes to label *TNFA* and *IL1B* mRNA. **(A)** Representative fluorescence microscopy picture of TNFA:A488 gene probe staining and hybridization series. **(B)** Representative fluorescence histogram for TNFA (FL1 INT) and IL1B (FL6 INT) co-hybridization. Percentage of *TNFA* mRNA abundance in **(C)** CD14^+^ cells and **(D)** in leukocytes. Percentage of *IL1B* mRNA abundance in **(E)** CD14^+^ cells and **(F)** in leukocytes. *n* = 10 cows per group. All data are given as LSM ± SEM. Different uppercase letters indicate trends 0.05 < *P* < 0.09.

Next, we analyzed the mRNA abundances of inflammatory cytokines and mediators in CD14^+^ monocytes and total leukocytes. The PrimeFlow analysis revealed no differences in the *TNFA* and *IL1B* (**Figures 3A–F**) or *IL6* and *INFG* (**Supplementary Figures S1B–D**) abundances in CD14^+^ cells or total leukocytes between the groups (*P* > 0.05). However, PF cows tended to have more leukocytic *IL1B* mRNA than CON cows on day 7 (*P* = 0.07; **Figure 3F**). Furthermore, the *IL6* and *IL1B* (*P* < 0.05) and *TNFA* (*P* = 0.054) mRNA abundances in leukocytes changed over time (**Figures 3D, F**, **Supplementary Figure S1C**).

### 3.5 RNAseq

**Figure 4:**
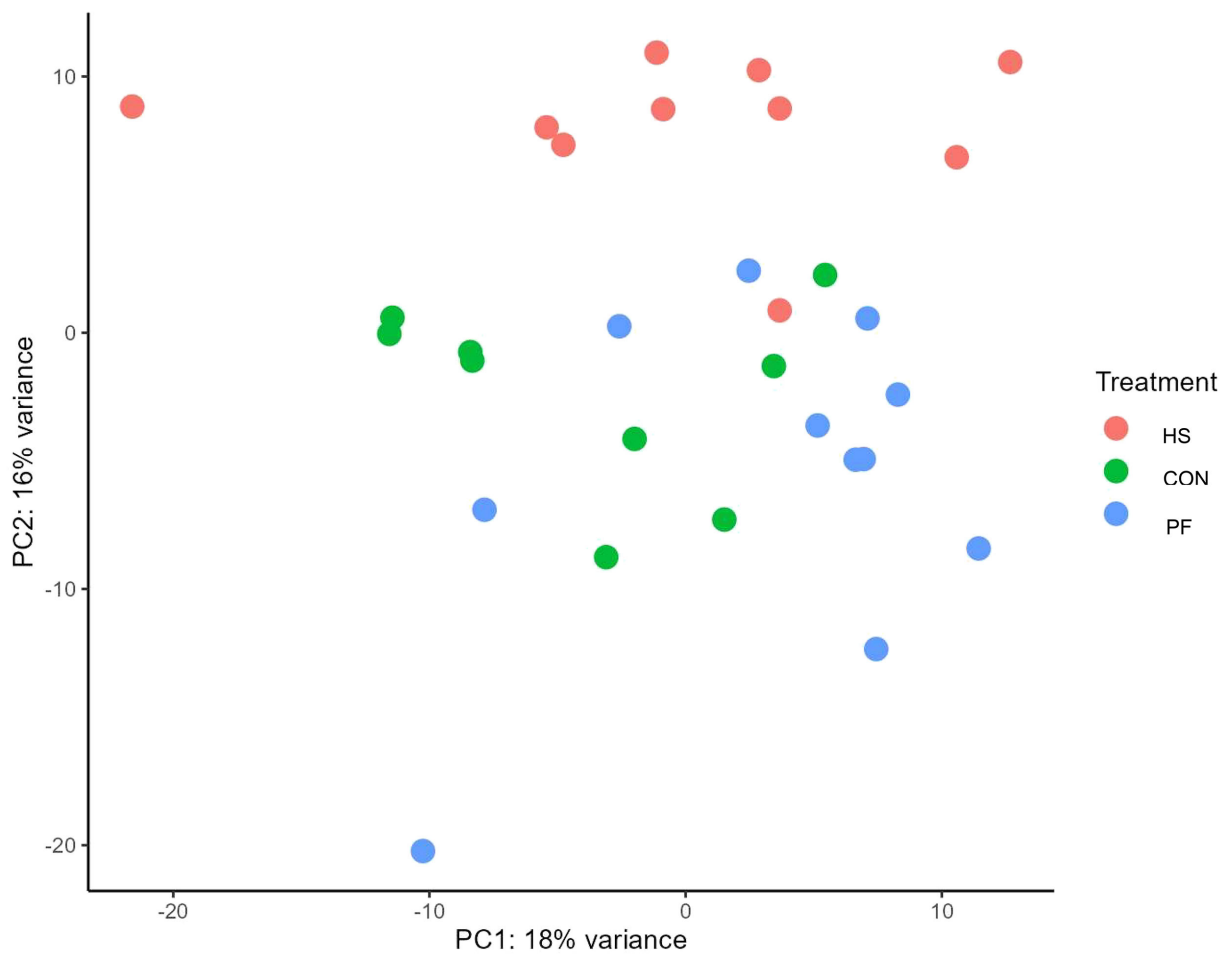
Sparse partial least squares-discriminant analysis (sPLS-DA) explains 18% of the variation 1 and 16% of the variation 2 (heat-stressed (HS; red), control (CON; green) and pair-fed (PF; blue) dairy cows) after 6 days of treatment.

To study further the cellular pathway activations of the adaptive and innate immune response on the mRNA level, RNAseq analysis in PBMC (including T cells, B cells, monocytes, dendritic cells, and natural killer cells) sampled on days 1 and 6 was conducted. Demultiplexing and filtering yielded 80–100 million reads per PBMC sample. Approximately 90% were mapped to the bovine genome. The result of the Sparse Partial Least Squares-Discriminant Analysis (sPLS-DA) on day 6 revealed a discrimination between the gene expression of each group and explained 18% of the variation by component 1 and 16% of the variation by component 2 (**Figure 4**). It is worth noting that on day 1, almost no difference on gene expression was found between cow groups, and only *KLRF2* was 23-fold more highly expressed in HS than CON cows (*q* < 0.001; **Supplementary Table S1**). However, the transcriptome analysis on day 6 revealed 305 differentially expressed genes between HS and CON cows (*q* < 0.05, **Supplementary Table S2**). Among these, 63 genes were more highly expressed and 242 genes were expressed lower in HS compared to CON cows (**Supplementary Table S2**). Comparing HS to PF cows, the transcriptomic analysis showed 879 differentially expressed genes (*q* < 0.05, **Supplementary Table S3**). In the HS group, 501 genes were more highly expressed and 378 genes were expressed lower than in the PF group (*q* < 0.05, **Supplementary Table S3**). The top five genes that were most significantly expressed higher in HS compared to CON cows were *RBPMS2* (2.3-fold), *GJA10* (2.0-fold), *P2PX1* (1.9-fold), *CDKN1A* (1.8-fold), and *CROT* (1.7-fold), whereas *COL5A3* (-3.2-fold), *FMO2* (-2.6-fold), *SCN11A* (-1.6-fold), *MS4A14* (-1.6-fold), and *LRRC63* (-1.5-fold) were expressed significantly lower in HS than CON cows (*q* < 0.05; **Supplementary Table S2**). Comparing HS with PF cows, the five highest upregulated genes in HS cow were *P2PX1* (2.4-fold), *RBPMS2* (2.3-fold), *CROT* (1.9-fold), *CDKN1A* (1.8-fold), and *MAP2* (1.6-fold), while *HBA* (-3.3-fold), *MTUS1* (-2.5-fold), *TENM4* (-2.2-fold), *COL5A3* (-1.9-fold), and *MYOM3* (-1.9-fold) were the genes with the lowest expression in HS than PF cows (**Supplementary Table S3**).

### 3.6 Functional enrichment analysis

**Figure 5:**
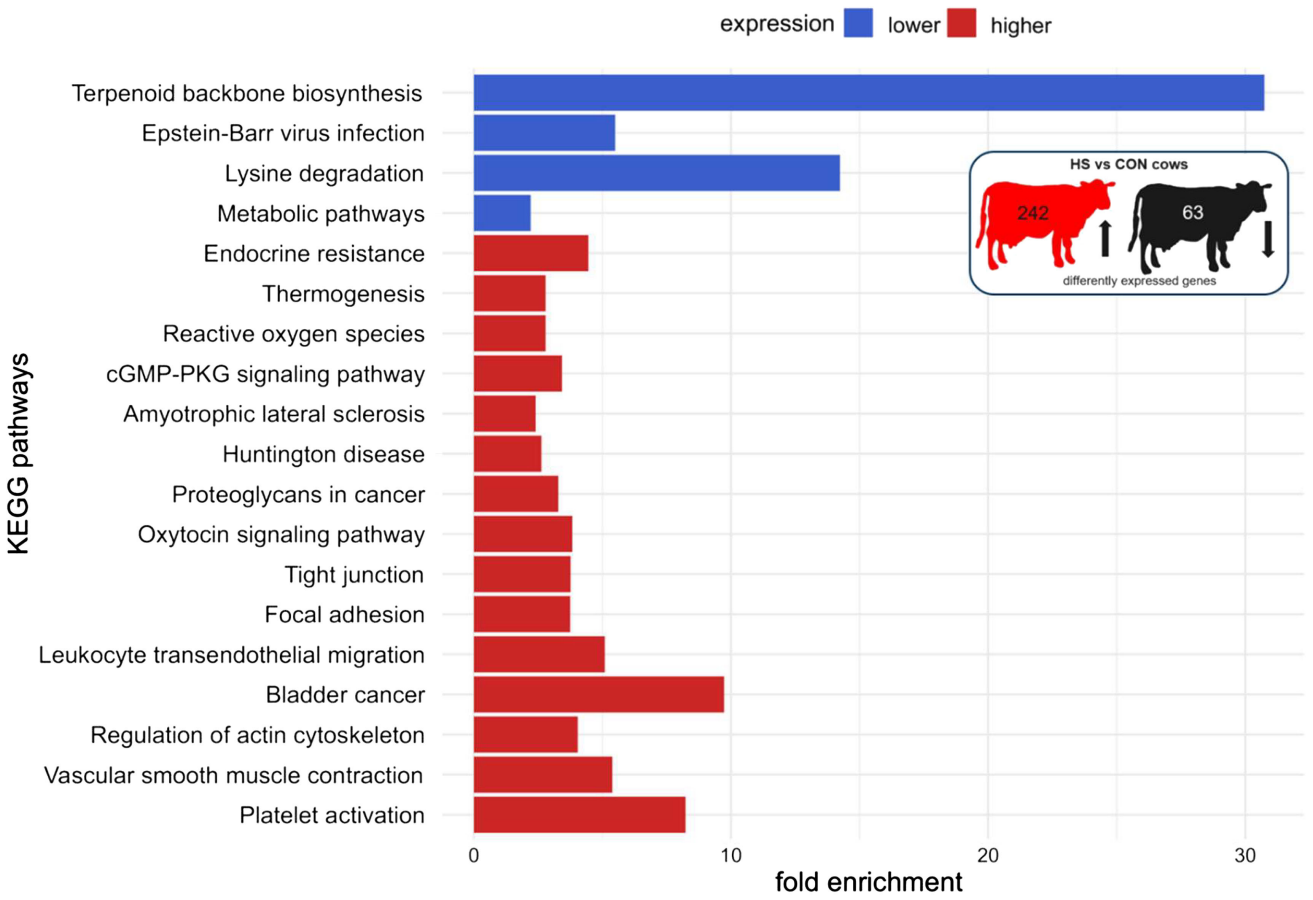
KEGG pathway enrichment analysis of mRNAs of PBMC after 6 days of heat-stressed (HS) and control (CON) dairy cows. The fold enrichment was determined as the ratio between the number of target genes assigned to a specific pathway and the total number of genes annotated to that pathway in the KEGG database. The bar chart indicates the fold enrichment of the top 15 pathways.

**Figure 6:**
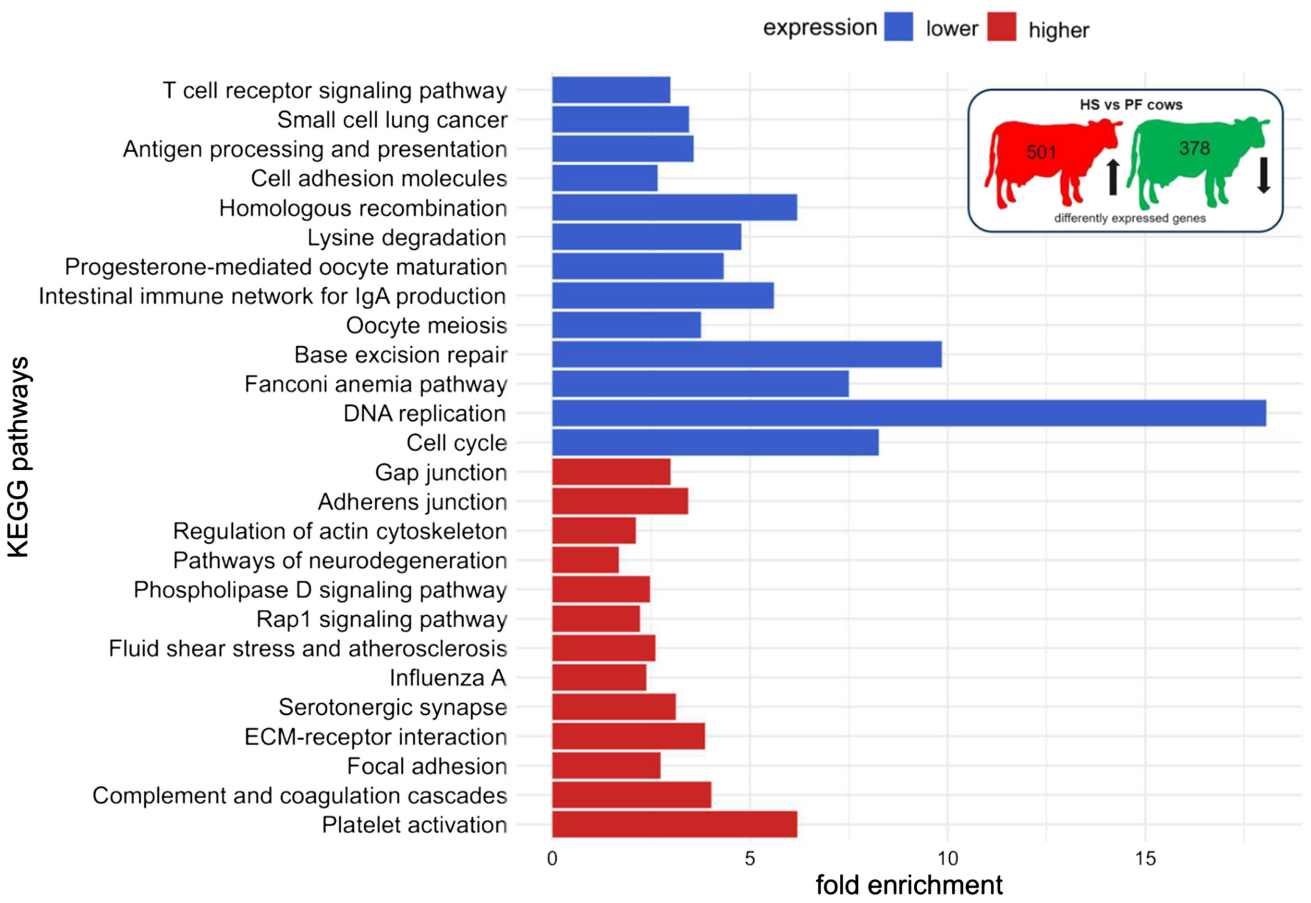
KEGG pathway enrichment analysis of mRNAs of PBMC after 6 days of heat-stressed (HS) and pair-fed (PF) dairy cows. The fold enrichment was determined as the ratio between the number of target genes assigned to a specific pathway and the total number of genes annotated to that pathway in the KEGG database. The bar chart indicates the fold enrichment of the top 15 pathways.

The functional pathway analysis in DAVID showed an enrichment of genes with higher expression of HS compared to CON cows in the following pathways: platelet activation, regulation of actin cytoskeleton, leukocyte transendothelial migration, focal adhesion, tight junction, oxytocin signaling pathway, cGMP-PKG signaling pathway, reactive oxygen species, thermogenesis, and endocrine resistance, whereas pathways related to terpenoid backbone biosynthesis, lysine degradation, and other metabolic pathways were enriched with genes that were expressed lower in HS compared to CON cows (**Figure 5**, **Supplementary Table S4**). The comparison of HS vs. PF cows revealed pathways enriched for genes encoding for platelet activation, complement and coagulation cascades, focal adhesion, ECM receptor, fluid shear stress and atherosclerosis, Rap1 signaling, phospholipase D signaling, regulation of actin cytoskeleton, and adherents junction, whereas genes with a lower expression were enriched in pathways regulating cell cycle, DNA replication, base excision repair, intestinal immune network for IgA production, lysine degradation, homologous recombination, antigen processing and presentation, and T cell receptor signaling (**Figure 6**, **Supplementary Table S5**).

## 4 Discussion

The primary objective of this study was to uncover the adaptive hematological and molecular mechanisms in blood and PBMC of heat-stressed dairy cows. In order to consider nutritional effects associated with the reduction in feed intake during heat stress, we used pair-feeding as an additional control besides *ad libitum* feeding at thermoneutrality to mimic the reduced nutrient availability under thermoneutral conditions and thus to distinguish between heat and nutritional stresses.

### 4.1 Impact of chronic heat stress on hematology profile, endotoxin, and LBP

In our study, heat stress had a stronger impact on the number of erythrocytes, hemoglobin, and hematocrit. The RBC, although still in the normal range with 6–8 × 10^6^ cells/µL, decreased after 7 days of heat stress and showed a trend toward lower hemoglobin and hematocrit in HS than in PF cows, suggesting the onset of normochromic normocytic anemia with unaltered MCH, MCHC, and MCV. Very limited information is available for heat stress effects on RBC-related parameters in mid-lactating cows. One study reported lower RBC, hematocrit, and RDW prepartum in summer-calving cows compared to winter-calving cows (11). In our study, lower serum iron concentrations were found in HS cows compared to both ambient temperature groups. These results are consistent with a previous study showing that lower hemoglobin concentrations and hematocrit were associated with lower serum iron concentrations in HS calves (42). In both studies, the decrease in erythrocytes, hemoglobin, and hematocrit was explained by a reduction in cellular oxygen demand and to reduce heat production due to oxidative metabolism (43). Different mechanisms are possible to explain the lower erythrocyte number occurring during heat stress. The lower RBC might be caused by a hemodilution effect, as more water is taken up and transported into the circulation to increase heat dissipation (11). Earlier findings from this heat stress trial reported a significant increase in water uptake per kilogram of dry matter intake after 2 days in HS compared to CON cows and after 5 days when compared to PF cows (33). Another mechanism might be that autoinflammation and subsequent hemolysis may reduce the lifespan of erythrocyte. The causes of autoinflammation are related to increased reactive radical formation and toxin activation (44). Furthermore, the accumulation of reactive oxygen species might damage the erythrocyte membrane and induce erythrocyte deformation, leading to erythrocyte dysfunction and removal from the blood circulation (45). Hypoxic episodes in the splanchnic area and diminished gut barrier function with ingress of toxic particles are likely to occur during heat stress (7, 46) and might enhance the hemolysis of erythrocytes. The lower erythrocyte number was found to be associated with lower serum iron concentration during heat stress, suggesting the disturbance of iron homeostasis accompanied with reduced iron availability for erythrocyte production (47). Under conditions of anemia in humans, iron is sequestered in ferritin within macrophages of the reticuloendothelial system, resulting in the reduced availability of iron-bound transferrin for erythropoiesis (48). However, this issue needs further research to elucidate the cellular mechanism in erythrocytes of heat-stressed dairy cows.

Interestingly, HS cows tended to have lower relative platelet and PCT and significantly lower MPV and PDW on day 6 compared to PF, but not CON cows. Different studies have reported that moderate and severe heat stress causes microvascular injuries of the epithelium, dominantly found in gut, kidney, and lungs (49, 50). Platelets and leukocytes adhere to the vascular endothelium and activate the coagulation pathway during severe heat stress (50). In our study with moderate heat stress, the temporary decline in the relative number of circulating platelets might be linked to the sealing of microvascular injuries in HS cows. However, it is not entirely clear why these differences did not occur between HS and CON cows, and thus it merits future research.

Our data showed that 7 days of persistent heat stress was associated with a dynamic lymphocyte response. Specifically, the absolute number of blood lymphocytes in HS cows decreased in a time-dependent manner and was lower than in PF cows but was not different from CON cows. This phenomenon started to occur after 3 days of heat stress and persisted until the end of the period, indicating that under conditions of long-, but not short-, term heat stress the number of lymphocytes declines. Previous results have shown a tendency for lower lymphocyte numbers in the prepartum and postpartum period in summer-calving cows to winter-calving cows (11). In buiatrics, a decline in the number of leukocytes is usually associated with stress, fear, exercise, or parturition, leading to lymphocytopenia (51). Possibly, the lower number of blood lymphocytes observed in HS compared to PF cows may be explained by heat-stress-induced lymphocytopenia, but this assumption needs to be proven in future investigations.

A lower leukocyte number in the blood circulation is associated with endotoxemia and inflammation (11). The presence of endotoxin in the systemic circulation might be the cause for a low-grade inflammation during mild heat stress as mentioned earlier. Daily endotoxin measurements revealed an initial increase of endotoxin after 1 day of heat stress, a decline between days 3 and 5, and a re-increase until 7 days of heat stress, indicating the dynamic adaptational processes of HS cows to maintain immune homeostasis. Interestingly, 7 days of pair-feeding increased the serum endotoxin concentrations in comparison to *ad libitum* feeding, suggesting that the reduction in feed intake is a sufficient criterion for the increase in endotoxin concentration. A previous study in pigs showed that both pair-feeding and heat stress increased the serum endotoxin levels after a short-term environmental challenge (12 h) compared to CON pigs (52). Pearce et al. (2015) related their results to a reduced gut integrity and/or liver capacity to detoxify endotoxin molecules (52). In dairy cows, a 60% reduction in feed intake for 5 days relative to *ad libitum* feeding increased the gut permeability, as indicated by increased plasma LBP and Cr concentrations upon oral Cr administration (53). An expected increase of the LBP, as observed after 14 days of heat stress in dairy cows (54), was not found in the present study, which might be due to individual differences among the animals.

### 4.2 Leukocytic NF-κB p65 signaling during chronic heat stress

To analyze how heat stress affects the response of the immune system, we studied the effect of chronic heat stress on the daily leukocytic NF-κB p65 and HSP70 presence potentially regulating the gene expression of pro-inflammatory cytokines. Our results showed a first drop of the mean NF-κB p65 FI on day 5 and a subsequent rise in HS cows compared to both control groups kept under thermoneutral conditions, indicating dynamic changes and a temporary higher NF-κB p65 presence in the nucleus, although the total number of NF-κB p65^+^ cells were maintained. Interestingly, HSP70 was not altered during heat stress, indicating that HSP70 does not appear to be involved in the regulation of NF-κB p65 translocation, which was not expected. The activation of the CD14/TLR2/4 pathways leads to the translocation of NF-κB p65 from the cytosol to the nucleus, a condition known to initiate, among others, the gene expression of pro-inflammatory cytokines (55). In this study, we measured in leukocytes and CD14^+^ cells concomitantly the gene expression of *TNFA*, *IFNG*, *IL1B*, and *IL6*. Unfortunately, the variation in gene expression among animals was enormous; therefore, we could not find significant differences between groups. The limited number of animals may be one reason for this. Thus, future experiments should be conducted with a larger sample size of animals to gain a more comprehensive understanding of the cytokine expression in CD14^+^ positive cells. However, in an earlier study using the same number of animal, we showed that heat stress induced an increase in plasma TNFα and IFNγ concentrations and *TNFA* mRNA abundance in the PBMC of HS cows (33), suggesting differences between the transcriptional and protein expression levels or a different origin of pro-inflammatory cytokines than from circulating leukocytes and CD14^+^ cells.

The transient activation of NF-κB p65 signaling was not found parallel in time with increased blood endotoxin concentration triggering the TLR2/4 pathways. However, other pathways, e.g., Notch, MAPK, and JAK-STAT, also influence NF-κB p65 activation and the transcription of cytokines, which may lead to higher plasma TNFα and IFNγ concentrations (56–59), particularly at the beginning of heat stress (33). Further research is required to gain detailed insight into the complex immune signaling system of heat-stressed dairy cows.

### 4.3 Adaptation of molecular, immune, and coagulation pathways to heat stress

We examined the immunological response in PBMC using RNA sequencing to improve our understanding of the molecular regulatory mechanism after short-term (24 h) and long-term (6 days) heat stress. Surprisingly, 24 h of heat stress did not alter the gene expression pattern in comparison to both control groups. Significant differences were found between the treatment groups after 6 days of chronic heat stress. The most prominent signaling pathway upregulated in HS than in both control groups was the activation of platelets in conjunction with vascular smooth muscle contraction, complement and coagulation cascade, and focal adhesion (**Table 3**). In line with this finding, earlier studies in different species described that heat stress leads to endothelial microvascular injuries, thrombosis, fibrinolysis, and inflammation, resulting in disseminated intravascular coagulation and compromised blood supply to organs (50, 60, 61). The microvascular injury might be caused by fluid sheer stress due to the increasing blood flow from the gut toward the skin as an attempt to minimize heat load (61). An activated endothelium attracts platelets, monocytes, and neutrophils which can initiate and amplify coagulation (50). For the activation of platelets, different sets of genes belonging to hemostasis were more highly expressed in HS cows (1): vasoconstriction (*VASP*) (2), platelet activation (*PTGR1*, *GNAS*, *P2X1*, *ITGA2*, and *ITGA2B*), and (3) coagulation cascade with clot formation (*VWF*, *F2RL3*, *F2*, *GP5*, and *GP1BA*). The required platelets for this process might also explain the tendency for the lower relative platelet count on day 6 of heat stress. An activated coagulation cascade including coagulation factors and fibrinogens was evidenced in the plasma proteome of lactating dairy cows sampled in summer compared to winter, which agrees with our findings (62). Furthermore, our enrichment analysis revealed the activation of leukocyte transendothelial migration during 6 days of heat stress. In PBMC, genes related to docking structure (*VASP*, *VCL*, and *ACT*) and F11 receptor (*F11R*) were found to be more highly expressed during heat stress. The F11 receptor is involved in T cell and neutrophil transmigration (63). The data imply leukocyte transendothelial migration via chemoattractants, e.g., pro-inflammatory cytokines (64) that may guide these immune cells to their destination. Recently published data from the same animals revealed higher plasma TNFα and IFNγ concentrations after 7 days of heat stress in comparison to CON and/or PF cows, suggesting an increased level of chemoattractants (33), which, in turn, supports our RNAseq data. However, future research needs to be intensified to prove altered coagulation by testing clotting time and platelet aggregation tests in HS cows under practical conditions.

**Table 3:** Summary of common major effects in heat-stressed cows either compared to control or pair-fed cows after 7 days of treatment.

Expression	Pathway	Implications
*Higher*	Activation of platelets	Platelet activation to repair endothelial microvascular injuries caused potentially by fluid sheer stress
	Complement and coagulation cascade	Coagulation cascade with clot formation (severe heat stress: thrombosis and fibrinolysis)
	Focal adhesion	Leukocyte transendothelial migration
	Vascular smooth muscle contraction	Vasoconstriction
*Lower*	Metabolic pathways	Adaptive metabolic mechanism to reduced feed and nutrient uptake or absorption

Among the pathways less activated in HS cows in comparison to both control groups were metabolic pathways (**Table 3**). Immune cells are able to sense metabolic stress and adapt adequately to new nutritional situations (65). Heat stress seems to affect the protein metabolism by the reduced expression of genes involved in lysine degradation (*SUV39H1*, *ALDH3A2*, *EZH2*, and *HYKK*). The altered mRNA expression of these genes could be caused by altered plasma lysine concentrations, which, unfortunately, were not analyzed in this study. Recently, we observed no difference in plasma lysine concentration between HS and PF cows (66), suggesting that plasma lysine concentrations did not affect the lysine degrading pathways in PBMC. Rather, the reduced expression of genes involved in lysine degradation seems to spare lysine for cellular protein synthesis.

Moreover, heat stress negatively affects the lipid metabolism of PBMC by reducing the mRNA expression of *ACAT2*, *CROT*, *MAPK14*, *SCL25A17*, and *SCD*. Several studies reported reduced plasma non-esterified fatty acids (NEFA) concentration under thermal stress (67, 68). There is increasing evidence that NEFA does not seem to be a desirable fuel for a heat-stressed organism, potentially to prevent metabolic heat production via β-oxidation in the mitochondria (66, 69).

Surprisingly, we did not find any enrichment of immune-response-related pathways in PBMC of HS cows in comparison to both control groups. However, only the comparison between HS and PF cows revealed a lower expression of genes involved in the T cell receptor signaling pathway (*CD28*, *ICOS*, *CD8A*, and *LOC100296565* (*T cell receptor alpha variable 18)*, *LOC100300510 (T cell receptor beta-1 chain C region*)), antigen processing and presentation (*CD8A*, *LOC100296565*, *LOC100300510*, *HSPA8*, and *NFYA*), and the intestinal immune network for IgA production (*CD28*, *ICOS*, *LOC100296565*, *LOC100300510*, and *AICDA*) in HS cows. The genes *CD28* and *Inducible T Cell Costimulator (ICOS*) belong to the same T cell surface receptor family. *ICOS* encodes an important T cell enhancer and is responsible for differentiation, proliferation, and cytokine production, mediates the interaction between T and B cells, and promotes antigen secretion by B cells (70). It is known that the differentiation of T cells into memory and effector cells is supported by metabolic pathways (71). A small portion of T cells differentiates into memory T cells that is primed by fatty acid oxidation and mitochondrial metabolism to sustain the energetic requirements (71). The lower activity of the lipid metabolism could provide fewer fatty acids to promote T cell differentiation, which could partially explain the inactivated T cell receptor signaling pathway of PBMC containing T cells and immune suppression during heat stress. Zachut et al. (2020) showed that summer-calving cows had less CD8^+^ cytotoxic T cells and CD335^+^ natural killer cells than winter-calving cows (11), which could also be a cause of lower proliferation capacity during heat stress (15). As a limitation of our study, we did not differentiate the PBMC population into B cells, T cells, and cytotoxic or natural killer cells. More research is required to distinguish between the different cell types and their function during heat stress to identify potential targets for the development of new treatment strategies.

## 5 Conclusion

In conclusion, chronic heat stress in mid-lactating, non-pregnant dairy cows induced dynamic changes of the blood profile by reducing erythrocytes, hemoglobin, hematocrit, serum iron concentration, platelets, and number of lymphocytes, suggesting the onset of normochromic normocytic anemia. Heat stress and high serum endotoxin concentrations might be transiently associated with NF-κB p65 signaling in peripheral blood leukocytes by enhancing the presence of NF-κB p65 in the nucleus. On the transcriptome level, the present study revealed an indication on the activation of vasoconstriction, platelet activation, and coagulation cascade with concomitant leukocyte migration in PBMC during heat stress. The latter was potentially caused by microvascular injuries and fluid sheer stress due to altered blood circulation from the splanchnic area toward the skin. Moreover, specific immune pathways related to T cell receptor signaling pathway, antigen processing and presentation, and intestinal immune network for IgA production and metabolic pathways related to lysine degradation and lipid metabolism were downregulated, which implies immunosuppression and metabolic adaptation of lymphocytes to lower nutrient availability during high ambient temperatures. The activation of blood coagulation and the onset of immunosuppression need to be studied in detail in future experiments in order to develop practical guidelines for veterinarians to maintain animal health during summer months. Furthermore, our present study emphasizes the importance of management tools mitigating heat stress on farms by applying cooling (e.g., shade, ventilation, and sprinklers), ensuring a sufficient water supply, and implementing nutritional interventions (e.g., electrolyte and feed additive supplementations, feeding during cooler periods of the day).

## Data Availability

The datasets presented in this study can be found in online repositories. (European Nucleotide Archive (project id: PRJEB88229; submission ID: ERA31968147).

## References

[1] IPCC. Summary for Policymarkers. In: Pötner R Tignor P Mintenbeck A Craig L Löschke M Okem R, editors. Climate Change 2022: Impacts, adaptation and vulnerability. Cambridges Universitiy Press, Cambridge, UK and New York, NY, USA (2022). Contribution of Working Group II to the Sixth Assessment Report of the Intergovernmental Panel on Climate Change. doi: 10.1017/9781009325844.001

[2] Faurie C Varghese BM Liu J Bi P. Association between High temperature and heatwaves with heat-related illnesses: A systematic review and meta-analysis. Sci Total Environ. (2022) 852:158332. doi: 10.1016/j.scitotenv.2022.158332, PMID: 36041616

[3] Sejian V Bhatta R Gaughan JB Dunshea FR Lacetera N. Review: adaptation of animals to heat stress. Animal. (2018) 12:s431–s44. doi: 10.1017/S1751731118001945, PMID: 30139399

[4] Fuquay JW. Heat stress as it affects animal production. J Anim Sci. (1981) 52:164–74. doi: 10.2527/jas1981.521164x, PMID: 7195394

[5] Bernabucci U Lacetera N Baumgard LH Rhoads RP Ronchi B Nardone A. Metabolic and hormonal acclimation to heat stress in domesticated ruminants. Animal. (2010) 4:1167–83. doi: 10.1017/S175173111000090X, PMID: 22444615

[6] Hall DM Baumgardner KR Oberley TD Gisolfi CV. Splanchnic tissues undergo hypoxic stress during whole body hyperthermia. Am J Physiol. (1999) 276:G1195–203. doi: 10.1152/ajpgi.1999.276.5.G1195, PMID: 10330010

[7] Lambert GP. Stress-induced gastrointestinal barrier dysfunction and its inflammatory effects. J Anim Sci. (2009) 87:E101–8. doi: 10.2527/jas.2008-1339, PMID: 18791134

[8] Mota-Rojas D Titto CG Orihuela A Martinez-Burnes J Gomez-Prado J Torres-Bernal F. Physiological and behavioral mechanisms of thermoregulation in mammals. Anim (Basel). (2021) 11(6):1733. doi: 10.3390/ani11061733, PMID: 34200650 PMC8227286

[9] Bohlouli M Yin T Hammami H Gengler N Konig S. Climate sensitivity of milk production traits and milk fatty acids in genotyped holstein dairy cows. J Dairy Sci. (2021) 104:6847–60. doi: 10.3168/jds.2020-19411, PMID: 33714579

[10] Bernabucci U Basirico L Morera P Dipasquale D Vitali A Piccioli Cappelli F. Effect of summer season on milk protein fractions in holstein cows. J Dairy Sci. (2015) 98:1815–27. doi: 10.3168/jds.2014-8788, PMID: 25547301

[11] Zachut M Kra G Nemes-Navon N Ben-Aharon N Moallem U Lavon Y. Seasonal heat load is more potent than the degree of body weight loss in dysregulating immune function by reducing white blood cell populations and increasing inflammation in holstein dairy cows. J Dairy Sci. (2020) 103:10809–22. doi: 10.3168/jds.2020-18547, PMID: 32896401

[12] Lamp O Derno M Otten W Mielenz M Nurnberg G Kuhla B. Metabolic heat stress adaption in transition cows: differences in macronutrient oxidation between late-gestating and early-lactating german holstein dairy cows. PloS One. (2015) 10:e0125264. doi: 10.1371/journal.pone.0125264, PMID: 25938406 PMC4418699

[13] Shaffer L Roussel JD Koonce KL. Effects of age, temperature-season, and breed on blood characteristics of dairy cattle. J Dairy Sci. (1981) 64:62–70. doi: 10.3168/jds.S0022-0302(81)82529-5, PMID: 7264021

[14] Elvinger F Hansen PJ Natzke RP. Modulation of function of bovine polymorphonuclear leukocytes and lymphocytes by high temperature *in vitro* and *in vivo* . Am J Vet Res. (1991) 52:1692–8. doi: 10.2460/ajvr.1991.52.10.1692, PMID: 1662922

[15] Lacetera N Bernabucci U Scalia D Ronchi B Kuzminsky G Nardone A. Lymphocyte functions in dairy cows in hot environment. Int J Biometeorol. (2005) 50:105–10. doi: 10.1007/s00484-005-0273-3, PMID: 15991017

[16] Ju XH Xu HJ Yong YH An LL Jiao PR Liao M. Heat stress upregulation of toll-like receptors 2/4 and acute inflammatory cytokines in peripheral blood mononuclear cell (Pbmc) of bama miniature pigs: an *in vivo* and *in vitro* study. Animal. (2014) 8:1462–8. doi: 10.1017/S1751731114001268, PMID: 24912383

[17] Olde Riekerink RG Barkema HW Stryhn H. The effect of season on somatic cell count and the incidence of clinical mastitis. J Dairy Sci. (2007) 90:1704–15. doi: 10.3168/jds.2006-567, PMID: 17369210

[18] Gao J Barkema HW Zhang L Liu G Deng Z Cai L. Incidence of clinical mastitis and distribution of pathogens on large chinese dairy farms. J Dairy Sci. (2017) 100:4797–806. doi: 10.3168/jds.2016-12334, PMID: 28434736

[19] Yeruham I Elad D Friedman S Perl S. Corynebacterium pseudotuberculosis infection in Israeli dairy cattle. Epidemiol Infect. (2003) 131:947–55. doi: 10.1017/s095026880300894x, PMID: 14596537 PMC2870040

[20] Cook NB Mentink RL Bennett TB Burgi K. The effect of heat stress and lameness on time budgets of lactating dairy cows. J Dairy Sci. (2007) 90:1674–82. doi: 10.3168/jds.2006-634, PMID: 17369207

[21] Castro-Montoya JM Gonzalez FL Mendoza MV Harper K Corea EE. Interrelationship between diseases and calving season and their impact on reproductive parameters and milk production of tropical dairy cows. Trop Anim Health Prod. (2022) 54:158. doi: 10.1007/s11250-022-03151-5, PMID: 35380316

[22] Waage S Sviland S Odegaard SA. Identification of risk factors for clinical mastitis in dairy heifers. J Dairy Sci. (1998) 81:1275–84. doi: 10.3168/jds.S0022-0302(98)75689-9, PMID: 9621229

[23] Morse D DeLorenzo MA Wilcox CJ Collier RJ Natzke RP Bray DR. Climatic effects on occurrence of clinical mastitis. J Dairy Sci. (1988) 71:848–53. doi: 10.3168/jds.S0022-0302(88)79626-5, PMID: 3372825

[24] Schneider PL Beede DK Wilcox CJ. Nycterohemeral patterns of acid-base status, mineral concentrations and digestive function of lactating cows in natural or chamber heat stress environments. J Anim Sci. (1988) 66:112–25. doi: 10.2527/jas1988.661112x, PMID: 3366700

[25] Vermunt JJ. Heat stress in dairy cattle – some potential health risks associated with the nutritional management of the condition. J Clin Veterinary Res. (2021) 1:1–6. doi: 10.54289/JCVR2100102

[26] Bagath M Krishnan G Devaraj C Rashamol VP Pragna P Lees AM. The impact of heat stress on the immune system in dairy cattle: A review. Res Vet Sci. (2019) 126:94–102. doi: 10.1016/j.rvsc.2019.08.011, PMID: 31445399

[27] Bradford BJ Yuan K Farney JK Mamedova LK Carpenter AJ. Invited review: inflammation during the transition to lactation: new adventures with an old flame. J Dairy Sci. (2015) 98:6631–50. doi: 10.3168/jds.2015-9683, PMID: 26210279

[28] Bertoni G Trevisi E. Use of the liver activity index and other metabolic variables in the assessment of metabolic health in dairy herds. Vet Clin North Am Food Anim Pract. (2013) 29:413–31. doi: 10.1016/j.cvfa.2013.04.004, PMID: 23809898

[29] Koch F Thom U Albrecht E Weikard R Nolte W Kuhla B. Heat stress directly impairs gut integrity and recruits distinct immune cell populations into the bovine intestine. Proc Natl Acad Sci U.S.A. (2019) 116(2):10333–8. doi: 10.1073/pnas.1820130116, PMID: 31064871 PMC6535017

[30] Pearce SC Mani V Weber TE Rhoads RP Patience JF Baumgard LH. Heat stress and reduced plane of nutrition decreases intestinal integrity and function in pigs. J Anim Sci. (2013) 91:5183–93. doi: 10.2527/jas.2013-6759, PMID: 23989867

[31] Mani V Weber TE Baumgard LH Gabler NK. Growth and development symposium: endotoxin, inflammation, and intestinal function in livestock. J Anim Sci. (2012) 90:1452–65. doi: 10.2527/jas.2011-4627, PMID: 22247110

[32] Medzhitov R. Toll-like receptors and innate immunity. Nat Rev Immunol. (2001) 1:135–45. doi: 10.1038/35100529, PMID: 11905821

[33] Koch F Otten W Sauerwein H Reyer H Kuhla B. Mild heat stress-induced adaptive immune response in blood mononuclear cells and leukocytes from mesenteric lymph nodes of primiparous lactating holstein cows. J Dairy Sci. (2023) 106:3008–22. doi: 10.3168/jds.2022-22520, PMID: 36894431

[34] Percie du Sert N Hurst V Ahluwalia A Alam S Avey MT Baker M. The arrive guidelines 2.0: updated guidelines for reporting animal research. PloS Biol. (2020) 18:e3000410. doi: 10.1371/journal.pbio.3000410, PMID: 32663219 PMC7360023

[35] Bharati J Dangi SS Mishra SR Chouhan VS Verma V Shankar O. Expression analysis of toll like receptors and interleukins in tharparkar cattle during acclimation to heat stress exposure. J Therm Biol. (2017) 65:48–56. doi: 10.1016/j.jtherbio.2017.02.002, PMID: 28343575

[36] Selkirk GA McLellan TM Wright HE Rhind SG. Mild endotoxemia, nf-kappab translocation, and cytokine increase during exertional heat stress in trained and untrained individuals. Am J Physiol Regul Integr Comp Physiol. (2008) 295:R611–23. doi: 10.1152/ajpregu.00917.2007, PMID: 18565834

[37] Weikard R Goldammer T Brunner RM Kuehn C. Tissue-specific mrna expression patterns reveal a coordinated metabolic response associated with genetic selection for milk production in cows. Physiol Genomics. (2012) 44:728–39. doi: 10.1152/physiolgenomics.00007.2012, PMID: 22669841

[38] Weikard R Goldammer T Eberlein A Kuehn C. Novel transcripts discovered by mining genomic DNA from defined regions of bovine chromosome 6. BMC Genomics. (2009) 10:186. doi: 10.1186/1471-2164-10-186, PMID: 19393061 PMC2681481

[39] Li H Handsaker B Wysoker A Fennell T Ruan J Homer N. The sequence alignment/map format and samtools. Bioinformatics. (2009) 25:2078–9. doi: 10.1093/bioinformatics/btp352, PMID: 19505943 PMC2723002

[40] Love MI Huber W Anders S. Moderated estimation of fold change and dispersion for rna-seq data with deseq2. Genome Biol. (2014) 15:550. doi: 10.1186/s13059-014-0550-8, PMID: 25516281 PMC4302049

[41] Huang da W Sherman BT Lempicki RA. Systematic and integrative analysis of large gene lists using david bioinformatics resources. Nat Protoc. (2009) 4:44–57. doi: 10.1038/nprot.2008.211, PMID: 19131956

[42] Trifkovic J Jovanovic L Bosnjakovic D Savic D Stefanovic S Krajisnik T. Summer season-related heat stress affects the mineral composition of holstein dams’ Colostrum, and neonatal calves’ Mineral status and hematological profile. Biol Trace Elem Res. (2022) 200:2122–34. doi: 10.1007/s12011-021-02834-8, PMID: 34286471

[43] Lee JA Roussel JD Beatty JF. Effect of temperature-season on bovine adrenal cortical function, blood cell profile, and milk production. J Dairy Sci. (1976) 59:104–8. doi: 10.3168/jds.S0022-0302(76)84163-X, PMID: 1249274

[44] Nairz M Theurl I Swirski FK Weiss G. Pumping iron”-how macrophages handle iron at the systemic, microenvironmental, and cellular levels. Pflugers Arch. (2017) 469:397–418. doi: 10.1007/s00424-017-1944-8, PMID: 28251312 PMC5362662

[45] Bettiol A Galora S Argento FR Fini E Emmi G Mattioli I. Erythrocyte oxidative stress and thrombosis. Expert Rev Mol Med. (2022) 24:e31. doi: 10.1017/erm.2022.25, PMID: 36017709 PMC9884766

[46] Lambert GP Gisolfi CV Berg DJ Moseley PL Oberley LW Kregel KC. Selected contribution: hyperthermia-induced intestinal permeability and the role of oxidative and nitrosative stress. J Appl Physiol (1985). (2002) 92:1750–61. doi: 10.1152/japplphysiol.00787.2001, PMID: 11896046

[47] Kim A Fung E Parikh SG Valore EV Gabayan V Nemeth E. A mouse model of anemia of inflammation: complex pathogenesis with partial dependence on hepcidin. Blood. (2014) 123:1129–36. doi: 10.1182/blood-2013-08-521419, PMID: 24357728 PMC9632791

[48] Schmidt PJ. Regulation of iron metabolism by hepcidin under conditions of inflammation. J Biol Chem. (2015) 290:18975–83. doi: 10.1074/jbc.R115.650150, PMID: 26055723 PMC4521019

[49] Bouchama A Bridey F Hammami MM Lacombe C al-Shail E al-Ohali Y. Activation of coagulation and fibrinolysis in heatstroke. Thromb Haemost. (1996) 76:909–15. doi: 10.1055/s-0038-1650685 8972010

[50] Roberts GT Ghebeh H Chishti MA Al-Mohanna F El-Sayed R Al-Mohanna F. Microvascular injury, thrombosis, inflammation, and apoptosis in the pathogenesis of heatstroke: A study in baboon model. Arterioscler Thromb Vasc Biol. (2008) 28:1130–6. doi: 10.1161/ATVBAHA.107.158709, PMID: 18388331

[51] Roland L Drillich M Iwersen M. Hematology as a diagnostic tool in bovine medicine. J Vet Diagn Invest. (2014) 26:592–8. doi: 10.1177/1040638714546490, PMID: 25121728

[52] Pearce SC Sanz Fernandez MV Torrison J Wilson ME Baumgard LH Gabler NK. Dietary organic zinc attenuates heat stress-induced changes in pig intestinal integrity and metabolism. J Anim Sci. (2015) 93:4702–13. doi: 10.2527/jas.2015-9018, PMID: 26523563

[53] Horst EA Mayorga EJ Al-Qaisi M Rodriguez-Jimenez S Goetz BM Abeyta MA. Evaluating effects of zinc hydroxychloride on biomarkers of inflammation and intestinal integrity during feed restriction. J Dairy Sci. (2020) 103:11911–29. doi: 10.3168/jds.2020-18860, PMID: 33041022

[54] Ruiz-Gonzalez A Rico DE Rico JE. Modulation of fecal metabolites by heat stress and diet, and their association with inflammation and leaky gut markers in dairy cows. Metabolites. (2022) 12(2):142. doi: 10.3390/metabo12020142, PMID: 35208216 PMC8874496

[55] Verstrepen L Bekaert T Chau TL Tavernier J Chariot A Beyaert R. Tlr-4, il-1r and tnf-R signaling to nf-kappab: variations on a common theme. Cell Mol Life Sci. (2008) 65:2964–78. doi: 10.1007/s00018-008-8064-8, PMID: 18535784 PMC11131687

[56] Sauma D Ramirez A Alvarez K Rosemblatt M Bono MR. Notch signalling regulates cytokine production by cd8+ and cd4+ T cells. Scand J Immunol. (2012) 75:389–400. doi: 10.1111/j.1365-3083.2012.02673.x, PMID: 22229688

[57] Obata T Brown GE Yaffe MB. Map kinase pathways activated by stress: the P38 mapk pathway. Crit Care Med. (2000) 28:N67–77. doi: 10.1097/00003246-200004001-00008, PMID: 10807318

[58] Hu Q Bian Q Rong D Wang L Song J Huang HS. Jak/stat pathway: extracellular signals, diseases, immunity, and therapeutic regimens. Front Bioeng Biotechnol. (2023) 11:1110765. doi: 10.3389/fbioe.2023.1110765, PMID: 36911202 PMC9995824

[59] Dahl GE Tao S Laporta J. Heat stress impacts immune status in cows across the life cycle. Front Vet Sci. (2020) 7:116. doi: 10.3389/fvets.2020.00116, PMID: 32211430 PMC7067922

[60] Bouchama A Roberts G Al Mohanna F El-Sayed R Lach B Chollet-Martin S. Inflammatory, hemostatic, and clinical changes in a baboon experimental model for heatstroke. J Appl Physiol (1985). (2005) 98:697–705. doi: 10.1152/japplphysiol.00461.2004, PMID: 15475604

[61] Burhans WS Rossiter Burhans CA Baumgard LH. Invited review: lethal heat stress: the putative pathophysiology of a deadly disorder in dairy cattle. J Dairy Sci. (2022) 105:3716–35. doi: 10.3168/jds.2021-21080, PMID: 35248387

[62] Min L Cheng J Zhao S Tian H Zhang Y Li S. Plasma-based proteomics reveals immune response, complement and coagulation cascades pathway shifts in heat-stressed lactating dairy cows. J Proteomics. (2016) 146:99–108. doi: 10.1016/j.jprot.2016.06.008, PMID: 27321583

[63] Czubak-Prowizor K Babinska A Swiatkowska M. The F11 receptor (F11r)/junctional adhesion molecule-a (Jam-a) (F11r/jam-a) in cancer progression. Mol Cell Biochem. (2022) 477:79–98. doi: 10.1007/s11010-021-04259-2, PMID: 34533648 PMC8755661

[64] Lopreiato V Vailati-Riboni M Parys C Fernandez C Minuti A Loor JJ. Methyl donor supply to heat stress-challenged polymorphonuclear leukocytes from lactating holstein cows enhances 1-carbon metabolism, immune response, and cytoprotective gene network abundance. J Dairy Sci. (2020) 103:10477–93. doi: 10.3168/jds.2020-18638, PMID: 32952025

[65] Amadori M Spelta C. The autumn low milk yield syndrome in high genetic merit dairy cattle: the possible role of a dysregulated innate immune response. Anim (Basel). (2021) 11(2):388. doi: 10.3390/ani11020388, PMID: 33546430 PMC7913622

[66] Koch F Lamp O Eslamizad M Weitzel J Kuhla B. D metabolic response to heat stress in late-pregnant and early lactation dairy cows: implications to liver-muscle crosstalk. PloS One. (2016) 11:e0160912. doi: 10.1371/journal.pone.0160912, PMID: 27513961 PMC4981427

[67] Kim WS Ghassemi Nejad J Peng DQ Jo YH Kim J Lee HG. Effects of different protein levels on growth performance and stress parameters in beef calves under heat stress. Sci Rep. (2022) 12:8113. doi: 10.1038/s41598-022-09982-4, PMID: 35581285 PMC9114135

[68] Gao ST Guo J Quan SY Nan XM Fernandez MVS Baumgard LH. The effects of heat stress on protein metabolism in lactating holstein cows. J Dairy Sci. (2017) 100:5040–9. doi: 10.3168/jds.2016-11913, PMID: 28390717

[69] Baumgard LH Rhoads RP Jr. Effects of heat stress on postabsorptive metabolism and energetics. Annu Rev Anim Biosci. (2013) 1:311–37. doi: 10.1146/annurev-animal-031412-103644, PMID: 25387022

[70] Honikel MM Olejniczak SH. Co-stimulatory receptor signaling in car-T cells. Biomolecules. (2022) 12(9):1303. doi: 10.3390/biom12091303, PMID: 36139142 PMC9496564

[71] Buck MD O’Sullivan D Geltink RIK Curtis JD Chang CH Sanin DE. Mitochondrial dynamics controls T cell fate through metabolic programming. Cell. (2016) 166:63–76. doi: 10.1016/j.cell.2016.05.035, PMID: 27293185 PMC4974356

