# Supplementary material for: Dynamic immune and molecular responses to chronic heat stress in blood and peripheral blood mononuclear cells of dairy cows
Source: Front Immunol. 2025 Sep 25;16:1633453. doi: 10.3389/fimmu.2025.1633453 (PMC12507569; doi:10.3389/fimmu.2025.1633453)
Supplement: Supplementary file 1 [file Table1.docx]

Supplementary Material

#
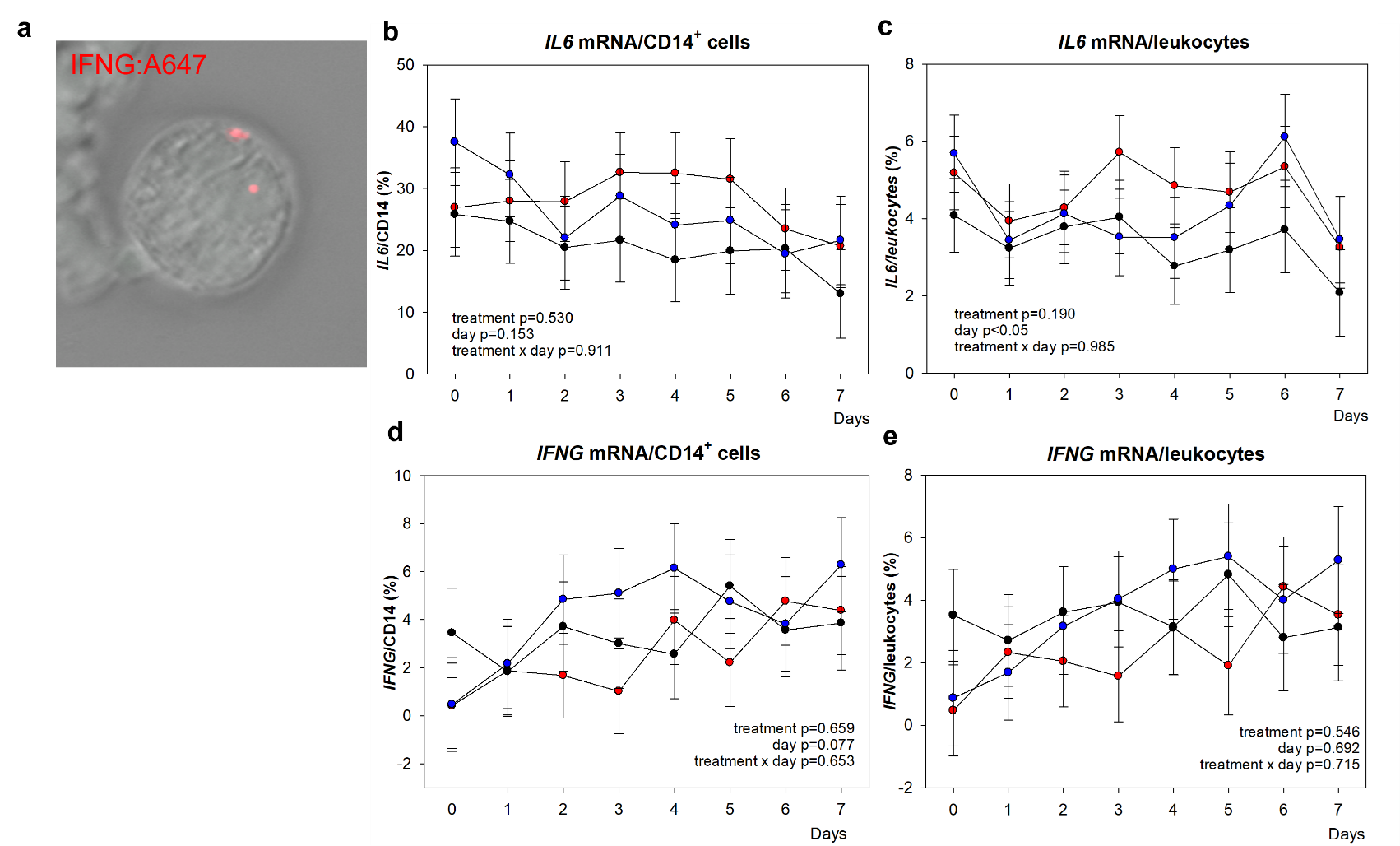
Supplementary Figures

**Figure S1.** *IL6* and *IFNG* mRNA abundances in blood leukocytes of heat-stressed (HS, red), control (CON, black) and pair-fed (PF, blue) cows determined by PrimeFlow RNA Assay. Leukocytes were stained with antibodies for CD14, fixed, permeabilized and hybridized with gene probes to label *IL6* and *IFNG* mRNA. (a) Representative fluorescence microscopy image of *IFNG*:A647 gene probe staining and hybridization series. (b) Portion of *IL6* mRNA abundance in (c) CD14^+^ cells and (d) in leukocytes. Portion of *IFNG* mRNA abundance in (e) CD14^+^ cells and (f) in leukocytes. n = 10 cows per group. All data are given as LSM ± SEM. Capital letter indicate trends (0.05 < PF < 0.09).

**
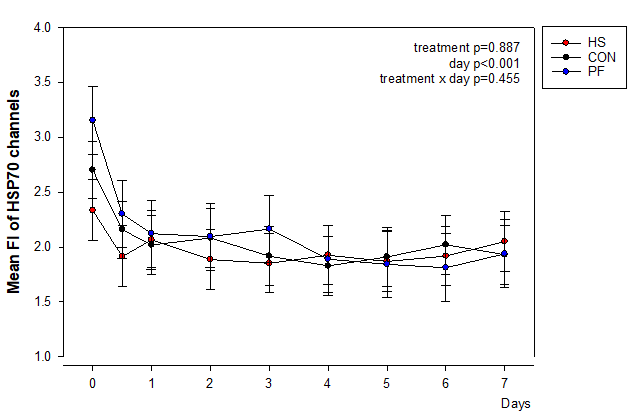
Figure S2.** Flowcytometrically analysis of heat shock protein 70 (HSP70) in leukocytes of heat-stressed (HS, red), control (CON, black) and pair-fed (PF, blue) dairy cows. Mean fluorescence intensity (FI) of HSP70 during 7 d of treatment. n = 10 cows per group. All data are given as LSM ± SEM.
